# Society Meetings

**Published:** 1894-05

**Authors:** 


### Society Meetings.

THE American Medical Publishers' Association will hold its annual meeting at White Sulphur Springs, West Virginia, Friday and Saturday, August 3 and 4, 1894. The annual dinner will be given in the evening of the first day. A number of practical papers are preparing, bearing upon subjects of interest to every one engaged in medical publishing. The above dates have been selected as a time when most business men can best spare a few days for a pleasure trip, and reduced rates having been secured at the hotels for members and their families, it is hoped that this meeting will be one of congeniality as well as of business interest. A special meeting has been called for June 4th, at San Francisco, in the Palace Hotel, at 1 P. M. sharp, for the transaction of special business. It is hoped that all publishers attending the A. M. A. will make it a point to be present at this meeting. Western and Southern members should write to E. B. Pope, C. & O. R'y, St. Louis, and arrange to advertise for transportation to White Sulphur Springs.

---

THE Roswell Park Medical Club gave its second annual dinner at the Tifft House, Buffalo, Thursday evening, April 12, 1894, at 9 o'clock. There were present Dr. J. H. Etheridge, of the Rush Medical College, Chicago; Drs. M. D. Mann, C. G. Stockton, Phelps, J. H. Potter, C. C. Fredericks, T. Bagley, Van Peyma, A. E. Collins, G. F. Cott, S. Y. Howell, M. A. Crockett, J. W. Putnam, F. S. Crego, J. A. Gibson, A. Lytel, DeLancey Rochester, Ross, Rev. H. A. Reed and Mr. A. L. Harrison.

Dr. J. C. Thompson acted as toastmaster, and short speeches were made by Drs. Roswell Park, J. H. Potter, J. H. Etheridge, G. F. Cott, C. G. Stockton and Rev. H. A. Reed. Dr. M. D. Mann read a paper on Ureteritis, which was discussed by Drs. Fredericks, Howell, Etheridge, Stockton, Crockett and others.

---

THE surgeons of the Western New York & Pennsylvania Railway Company met at Buffalo, April 5, 1894, and organized the Association of Western New York & Pennsylvania Railway Surgeons, electing the following officers: President, C. M. Daniels, M. D., Buffalo, N. Y.; vice-president, J. A. Ritchey, M. D., Oil City, Pa.; secretary and treasurer, E. M. Dooley, M. D., Buffalo, N. Y. The surgeons who were present expressed themselves unanimously in favor of the movement, and numerous letters were received from those who were unable to attend, giving every assurance that the association will be a success, while the management of the railroad most heartily indorsed the movement.

---

THE American Surgical Association will hold its annual meeting in the lecture room of the medical department of Columbia College, 1325 H. street, N. W., Washington, D. C., May 29, 30, 31 and June 1, 1894. Special subjects announced for discussion are:

I. The Surgical Treatment of Empyema, by John Ashurst, Jr., M. D.

II. Methods of Teaching Surgery, by J. S. Billings, M. D.

III. The Surgery of the Kidney, by L. M. Tiffany, M. D.

IV. Methods of Controlling Hemorrhage in Amputation at the Shoulder, as Illustrated by Amputation at the Shoulder-joint and of the Entire Upper Extremity, by W. W. Keen, M. D.

Papers are also announced by Hunter McGuire, M. D., and Joseph Ransohoff, M. D.

---

The Association of Military Surgeons of the United States will hold its annual meeting at Washington, May 1, 2 and 3, 1894. Dr. A. H. Briggs, of Buffalo, chairman of the committee on transportation, has obtained concessions in fares for persons attending this meeting from all the railways east of the Rocky mountains. A large attendance is expected.

---

At the second quarterly meeting of the Cleveland Medical Society, to be held Friday evening, June 22, 1894, Dr. William Pepper, late provost of the University of Pennsylvania, will deliver an address. Dr. Pepper will also hold a clinic the following morning at one of the hospitals, which the profession generally will be invited to attend.

---

The National Association of Railway Surgeons will hold its seventh annual meeting in Harmony Hall, Galveston, Texas, May 8, 9, 10 and 11, 1894, under the presidency of Surgeon W. J. Gilbraith, of Omaha, Neb. An elaborate preliminary program has been issued, and a large attendance is anticipated.

---

The American Dermatological Association will hold its eighteenth annual meeting at the Arlington Hotel, Washington, May 29, 30, 31 and June 1, 1894, under the presidency of Dr. R. B. Morrison, of Baltimore.

